# Prevalence, determinants and coercive strategies relating to marital rape among women in Oyo State, Nigeria

**DOI:** 10.4314/gmj.v56i2.9

**Published:** 2022-06

**Authors:** Akintayo O Ogunwale, Rotimi F Afolabi

**Affiliations:** 1 Department of General Studies, Oyo State College of Agriculture and Technology, Igboora, Oyo State, Nigeria; 2 Department of Epidemiology and Medical Statistics, Faculty of Public Health, College of Medicine, University of Ibadan, Nigeria

**Keywords:** rape, sexual violence, prevalence, women, Nigeria

## Abstract

**Objectives:**

The study assessed the prevalence, determinants and coercive strategies relating to Marital Rape (MR) among women in Oyo State, Nigeria.

**Design:**

A cross-sectional survey

**Setting:**

The study was conducted in 120 communities, 30 wards, 6 Local Government Areas in Oyo State.

**Participants:**

A total of 1200 ever-married women aged 18–60 years were recruited using a multi-stage sampling.

**Methods:**

The participants were interviewed using a semi-structured questionnaire which captured MR-related experiences and coercive strategies. Data were analysed using descriptive statistics, Chi-square, and logistic regression. Odds ratios (OR) and their corresponding 95% Confidence Interval (CI) were reported.

**Results:**

Respondents' mean age was 36.6±9.6 years, while husbands' mean age was 42.1±10.3 years. Most (n=1094, 91.2%) respondents had ever married once and 82.8% (n=993) were currently married. The prevalence of MR was 15.3%. Physical force (n=153, 25.9%), followed by threat (n=139, 23.5%) topped the list of coercive strategies involved in MR. Currently, married women were at a higher risk of MR (OR: 2.73, CI: 1.39–5.37, P = 0.04) relative to divorced women. Respondents whose husbands were aged <30 years were at lower risk of MR (OR:0.03, CI: 0.002–0.47, P=0.01). Women who take decisions on sex alone were more likely to suffer MR (OR:3.95, CI: 1.38 — 11.31, P=0.01).

**Conclusions:**

Marital rape existed among women with increased risk among those who were currently married, married to older partners or sole decision-makers on sex. Physical force was the commonest coercive strategy used to facilitate MR. Community-based MR -related interventions are recommended.

**Funding:**

None declared

## Introduction

Rape and other sexually violent behaviours are ranked among the most serious forms of gender-based violence affecting women in different parts of the world.^1^–^3^ According to the World Health Organization report, one-fifth of all women have experienced rape and other forms of related sexual assaults^3^. Women are disproportionally affected by rape and other forms of sexually violent behaviours.^1^–^4^

Rape can be defined as any form of sexual penetration which is often characterized by using force, threat, fear of harm or any other means to compromise someone's consent to indulge in sexual activity.^4^,^5^

It has been noted by several investigators^6^–^8^ that most perpetrators of rape are known to rape survivors; they could be strangers, acquaintances, friends, neighbours, dating partners or even spouses. Rape committed by a spouse or ex-spouse falls under the category of marital rape.^6^

Marital rape or spousal rape can be described as forceful sexual intercourse without spousal consent or free will, often characterized by the use of coercive or manipulative means.^6^,-^9^ Marital rape is a condemnable act that is frowned upon by several human right-related treaties or declarations.^11^,^12^ In Nigeria, the Violence Against Persons Prohibition Act that came into existence in 2015 prohibits the act of marital rape. Despite the legal frame-work, marital rape, unlike other forms of rape, has not received much public attention.

The occurrence of marital rape cuts across developed and developing nations.^11^,^12^ Even though either spouse could be a victim of marital rape, women are most affected.^1^–^4^ It has been noted that marital rape accounts for about 25% of all rapes and 10–14% of married women experience marital rape.^13^ Studies^15^–^17^ have also noted that marital rape is a pervasive health burden among many women in Nigeria. For instance, a study conducted in Imo State, Nigeria showed that 14.2% of pregnant women experienced forced sexual intercourse at the hands of their husbands.^15^

Marital rape affects women physically, emotionally, and mentally, and has a significant negative impact on families and society in general.^6^,^18^ Many survivors of marital rape are likely to experience repeated episodes of rape.^5^,^6^ Unfortunately, survivors of marital rape are less likely to get help or support from friends, family, crisis centres and the police than those who are survivors of other forms of rape.^19^ In Nigeria, lack of support may be attributed to the notion that sexual intercourse perpetrated by one's husband under duress does not fall into the socio-cultural definition of rape.

In Nigeria, there is a dearth of evidence-based data on women's marital rape-related experiences which can serve as useful baseline data for designing and implementing appropriate prevention and control interventions as well as contribute to monitoring progress towards the Sustainable Development Goal (SGD) 5.2. Specifically, the exact prevalence and associated determinants of mar-ital rape issues in several parts of Nigeria including Oyo State remain to be fully explored. There is therefore a need to explore the prevalence, determinants and coercive strategies relating to marital rape among women in Oyo State, Nigeria.

## Methods

### Study setting

This cross-sectional study was conducted between March and April 2018 in Oyo State, Nigeria. Oyo State, which has 33 Local Government Areas (LGAs), is the fifth most populated state in Nigeria and the second most populated state in the southwest geopolitical zone of Nigeria. According to the National Population Commission (NPC) records^20^, Oyo State population for 2017 was projected at 7,594,147 with female population constituting 49.9% (3,789,479). The NPC projection for 2017 revealed that there were 1,623,440 females that were ever-married in Oyo State.^20^ A further breakdown of the marital status of these women showed that those that were married, separated, divorced, and widowed were 1,485,753; 31,624; 15,454 and 90,609 respectively.^20^

Oyo State is predominantly inhabited by the Yoruba ethnic group. Nigeria is a low-middle-income country and Oyo State, where the study was carried out, is a less industrialised state.

The economy of Oyo State is based chiefly on agriculture and handicrafts. In addition, there are some residents who are involved in petty trading, self-employed businesses, civil service and corporate works. At the time of the study, the national minimum monthly wage for workers, which is mostly being implemented in government works, was ₦18,000 naira (the equivalent of 50 United State of America dollars).

### Study population, sample size and sampling technique

The study population comprised all categories of women with marital experience aged 18–60 years (including those who were currently married, divorced or widowed at the time of the study). The sample size for the survey was 1,200 women.

A multi-stage sampling procedure was used in selecting the study participants. The first stage involved the random selection of two LGAs from each of the three senatorial districts in the State using the balloting procedure. Of the 33 LGAs in Oyo State, Akinyele, Oyo West, Ogbomoso South, Ogbomoso North, Ibadan North, and Ibadan North East LGAs were randomly selected. The second stage entailed the selection of five wards from each of the six LGAs, resulting in a total of 30 wards selected using a simple random sampling technique. At the third stage, four communities were selected from the lists of communities in each of the selected 30 wards using a systematic sampling method, resulting in a total of 120 communities. Lists of the communities were obtained from the records of the Independent National Electoral Commission.^21^ The fourth stage involved the selection of 10 houses/compounds, where women who were ever-married lived, in each of the selected communities. A total of 1,200 houses were selected from all the communities involved using systematic random sampling. The list of houses compiled by NPC was used as the sampling frame. In case such information was not available, an enumeration of a number of compounds/houses in a sub-area/street was carried out to have a provisional sampling frame.

In the last stage, due to the stigma-inducing nature of rape, only one participant was interviewed per house to ensure confidentiality and undue sensitization of the study population. However, where two or more eligible women were met in a selected house, balloting was used to pick one of them for the interview.

### Instrument and data collection procedure

An interviewer-administered semi-structured questionnaire was used for data collection. The questionnaire was pre-tested among women in Osun State (a neighbouring State) whose socio-demographic characteristics were similar to those of the study participants. The questionnaire covered various issues including respondents' socio-demographic information and their partners' characteristics, respondents' sexual behaviours and history of sexual violence as well as questions on marital rape experiences, coercive strategies and/or situations that were used to facilitate marital rape. Marital rape was operationally defined as forced or coerced sexual intercourse experienced in the hands of a husband or ex-husband. The concept of marital rape in this study covers all forms of sexual intercourse such as vaginal, anal, and oral sexual intercourse. The prevalence of marital rape was measured in terms of “ever experienced”.

The administration of the questionnaire was achieved through the help of twelve Research Assistants (RAs). The RAs who were all married women and had postgraduate degrees (of public health, medical sociology, guidance and counselling) had previously participated in sexual violence-related studies. The RAs had a six-hour intensive training prior to the commencement of the study. They were trained on the use of the English and Yoruba versions of the questionnaire. The respondents were informed of the confidentiality of the interview process, voluntary participation, benefits of participating in the study and the inconvenience that might be experienced during the interview. The interviews were conducted on a one-on-one basis in comfortable places within participants' houses/compounds such as lobby, shops, and backyards or in other neighbourhood convenient places such as community halls. No one apart from the research team members was with the participants during the period of the interviews. All interviews were mainly conducted at the time most women were less busy between mid-day and afternoon (12noon — 4 pm). And each of the interviews lasted between 15–25 minutes. After being interviewed, counselling tips, useful health education messages, and referral advice were made available to some survivors of marital rape who desired it.

### Data Analysis

Each questionnaire was reviewed, cleaned, edited and properly coded. Statistical Package for Social Sciences (SPSS) version 20.0 was used to facilitate data analysis. Descriptive statistics, Chi-square and logistic regression methods were used for data analysis.

The variables that were significantly related to marital rape at the bivariate level (p<0.05) were included in adjusted logistic regression analysis at the multivariate level. Odds ratios (OR) and their corresponding 95% confidence interval (CI) were reported at a 5% level of significance.

### Ethical approval

The Oyo State Ministry of Health Ethics Review Committee approved the study protocol with approval number (Ref: AD13/479/065). Prior to the data collection, participants gave written informed consent and were informed of their freedom to withdraw from the interview at any point. Asides from collecting no identifying information from participants, only investigators and authorised research staff had access to the study questionnaires.

## Results

### Respondents' socio-demographic characteristics and partners' information

The socio-demographic characteristics of respondents are presented in Table 1.

**Table 1:** Socio-demographic characteristics of respond-ents (N=1200)

Characteristics	n (%)
**Age (in years)**	
**≤24**	57 (4.8)
**25 – 49**	977 (81.4)
**≥50**	166 (13.8)
**Mean ± sd**	36.6 ± 9.6
**Religion**	
**Christian**	780 (65.0)
**Islam**	394 (32.8)
**Traditional African Religion**	26 (2.2)
**Ethnicity**	
**Yoruba**	921 (76.8)
**Non-Yoruba**	279 (23.2)
**Level of Education**	
**No formal Education**	89 (7.4)
**Primary Education**	109 (9.1)
**Secondary Education**	495 (41.3)
**Tertiary Education**	507 (42.3)
**Marital Status**	
**Currently married**	993 (82.8)
**Widow**	88 (7.3)
**Divorce**	119 (9.9)
**Family type (of present or last relationship)**	
**Monogamy**	998 (83.2)
**Polygamy**	202 (16.8)
**Number of husbands ever married**	
**One**	1094 (91.2)
**Two**	94 (7.8)
**Three**	12 (1.0)
**Mean ± sd**	1.1 ±0.29
**Duration of marriage**	
**≤10 years**	703 (58.6)
**11 – 20 years**	302 (25.2)
**≥20 years**	195 (16.3)
**Mean ± sd**	11.7 ± 8.8
**Employment Status**	
**Working/Employed**	1010 (84.2)
**Not working/Unemployed**	190 (15.8)
Income level*+	
**<18,000 naira**	296 (29.3)
**18,001 – 35,999 naira**	480 (47.5)
**≥ 36, 000**	232 (23.2)
**Median**	25,000
**Respondents' use of alcohol**	
**Yes**	97 (8.1)
**No**	1103 (91.9)
**Respondents' use of other psychoactive** **drugs+**	
**Yes**	62 (5.2)
**No**	1103 (9.9)
**Partner's age (in years)+#**	
**<30 years**	53 (4.8)
**30 – 59 years**	977 (81.4)
**≥60 years**	83 (13.8)
**Mean ± sd**	42.1 ± 10.3
**Partner's Level of Education#**	
**At most primary**	110 (9.2)
**Secondary**	384 (32.0)
**Tertiary**	706 (58.8)
**Partner's use of alcohol#**	
**Yes**	448 (37.3)
**No**	752 (62.7)
**Partner's use of psychoactive drugs#**	
**Yes**	47 (3.9)
**No**	1153 (96.1)

n – number of subjects per group of characteristics

+ missing value not reported

sd – standard deviation

* 360 naira = 1 US dollar as at 2018 when the study was conducted

# present husbands or most recent hus-bands (in the case of divorcees and widows)

The respondents' mean and median age was 36.6 ± 9.6 years and 35 (18–60) years, respectively. Many women were aged 25 — 49 years (n=977, 81.4%), Christians (n=780, 65.0%), Yorubas (n=921, 76.8%), employed (n=1010, 84.2%), and currently married (n=998, 82.8%). Most women (n=1,094, 91.2%) were ever married once, while 507 (42.3%) women had tertiary education. More than a quarter (n=296, 29.3%) of the women earned below 18,000 naira monthly. The respondents' husbands' mean age was 42.1 ± 10.3 years and a median of 40 (20–85) years. More than half (n=706, 58.8%) of the respondents' husbands had tertiary education. About 37.3% (n=448) of the women reported that their husbands indulge in alcohol consumption.

### Information relating to respondents' sexual behaviour and history of sexual violence

Table 2 shows respondents' information relating to sexual behaviours and previous history of violence. The majority of the respondents had always taken joint decisions on sexual intercourse with their husbands (n=768, 64.0%), and had never declined their husbands' sexual requests or advances (n=913, 77.5%). Menstruation (n=670, 34.1%) topped the lists of the reasons for declining husbands' sexual request, followed by ill-health/sickness (n=354, 18.0%) and fatigue (n=323, 16.4%). Only 187 (15.6%) women had never fought with their husbands. One hundred and seven respondents (8.9%) had a history of sexual violence before being married.

**Table 2:** Respondents' information relating to sexual be-haviours and previous history of violence

N=1200	
Characteristics	n (%)
**Person(s) who take decision on sexual intercourse** #	
Self alone	121 (10.1)
Husband alone	311 (25.9)
Self and husband	768 (64.0)
**Refuse/decline husband's sexual request or advances** + #	
Yes	265 (22.5)
No	913 (77.5)
**Conditions or reasons for declining sexual intercourse** **with husband**#*	
Menstrual Period	670 (34.1)
Not feeling fine	354 (18.0)
Overwork/fatigue	323 (16.4)
Lack of interest	241 (12.3)
During disagreement	182 (9.3)
Tired of sex	145 (7.4)
Attempt to use force to have sex	48 (2.4)
^µ^Others	3 (0.3)
**How often respondent fight with husband** #	
Often	396 (33.0)
Sometimes	617 (51.4)
Never	187 (15.6)
**Age at sexual debut**	
≤14 years	53 (4.4)
15 – 19 years	430 (35.8)
≥20 years'	717 (59.8)
Mean ± sd	20.2 ± 3.4)
**Previous history of sexual violence (before marriage)** +	
Yes	107 (8.9)
No	1093 (91.1)

n –– number of subjects per group of characteristics

# present husbands or most recent husbands (in the case of divorcees and widows)

+ missing value not reported

* multiple responses reported

### Association between background characteristics and marital rape

Overall, 184 (15.3%) of the respondents had ever experienced marital rape. The Association of marital rape with different background characteristics is presented in Table 3.

**Table 3:** Distribution of marital rape by women and their spouses' characteristics

Characteristics	Marital rape		Chi-squared (χ2)	p-value
	Ever – n (%)	Never – n (%)		
**Women age (in year)**			12.80	0.002*
**≥24**	15 (26.3)	42 (73.7)		
**25 – 49**	156 (16.0)	821 (84.0)		
**≥50**	13 (7.8)	153 (92.2)		
**Marital Status**			22.65	<0.001*
**Currently married**	136 (13.7)	857 (86.3)		
**Widow**	12 (13.6)	76 (86.4)		
**Divorce**	36 (30.3)	83 (69.7)		
**Family type**			2.26	0.830
**Monogamy**	146 (14.6)	852 (85.4)		
**Polygamy**	38 (18.8)	164 (81.2)		
**Level of Education**			5.73	0.060
**At most primary**	34 (17.2)	164 (82.8)		
**Secondary**	87 (17.6)	408 (82.4)		
**Tertiary**	63 (12.4)	444 (87.6)		
**Employment Status**			0.17	0.38
**Working/Employed**	153 (15.1)	857 (84.9)		
**Not working/Unemployed**	31 (16.3)	159 (83.7)		
**Income level (in Naira)**			9.89	0.007*
**<18,000 naira**	49 (16.6)	247 (83.4)		
**18,001 – 35,999 naira**	82 (17.1)	398 (82.9)		
**≥ 36, 000**	21 (9.0)	213 (91.0)		
**Use of alcohol**			19.77	<0.001*
**Yes**	30 (30.9)	67 (69.1)		
**No**	154 (14.0)	949 (86.0)		
**Use of psychoactive drugs**			17.31	<0.001*
**Yes**	21 (33.9)	41 (66.1)		
**No**	163 (14.3)	975 (85.7)		
**Number of husbands ever** **married**			12.53	<0.001*
**1**	155 (14.2)	938 (85.8)		
**≥2**	29 (27.1)	78 (72.9)		
**Partner's age (in years)** ^+^			17.63	<0.001*
**<30 years**	15 (28.3)	38 (71.7)		
**30 – 59 years**	155 (15.9)	822 (84.1)		
**≥60 years**	2 (2.4)	81 (97.6)		
**Partner's Level of Education**			7.21	0.027*
**At most primary**	22 (20.0)	88 (80.0)		
**Secondary**	70 (18.2)	314 (81.8)		
**Tertiary**	92 (13.0)	614 (87.0)		
**Partner's use of alcohol**			46.8	<0.001*
**Yes**	110 (24.6.)	338 (75.4)		
**No**	74 (9.8)	678 (90.2)		
**Partner's use of psychoactive** **drugs**			16.36	<0.001*
**Yes**	17 (36.2)	30 (63.8)		
**No**	167 (14.5)	986 (85.5)		
**Take decision relating to** **sex**			57.34	<0.001*
**Self alone**	11 (9.1)	110 (90.9)		
**Husband alone**	89 (28.6)	222 (71.4)		
**Self and husband**	84 (10.9)	684 (89.1)		
**History of sexual violence** **(before marriage)**			221.77	<0.001*
**Yes**	69 (65.1)	37 (34.9)		
**No**	115 (10.5)	979 (89.5)		
**Respondent fight with** **husband**			34.45	<0.001*
**Often**	53 (28.3)	134 (71.7)		
**Sometimes**	93 (15.1)	524 (84.9)		
**Never**	38 (9.6)	358 (90.4)		

* Significant at 5%

n – number of subjects

The prevalence of marital rape among respondents aged 18–24, 25–49 and 50–60 years were 26.3% (n=15), 16.0% (n=156), and 7.8% (n=13) respectively. Nearly all the selected variables had a significant (p<0.05) association with marital rape except for family type, women's level of education and women's employment status (see Table 4). A significantly higher prevalence of marital rape (n=30, 30.9%) was noted among women who indulged in the use of alcohol, while 33.9% (n=21) was observed among those who used psychoactive drugs. A remarkable higher prevalence of marital rape (n=69, 65.1%) was found among women who had a previous history of sexual victimization.

**Table 4:** Adjusted odds ratio of factors associated with marital rape

Characteristics	Adjusted odds ratio (aOR)	95% CI	p-value
**Women age (in years)**			
**≤24**	0.97	0.21 – 4.48	0.97
**25 – 49**	1.2	0.43 – 3.31	0.74
**≥50 (Ref)**	1		
**Marital Status**			
**Currently married**	2.68	1.32 – 5.41	0.006*
**Widow**	NA	NA	NA
**Divorce (Ref)**	1		
**Income level (in Naira)**			
**<18,000**	0.68	0.33 – 1.39	0.29
**18,001 – 35,999**	0.64	0.34 – 1.20	0.16
**≥ 36, 000 (Ref)**	1		
**Use of alcohol**			
**Yes**	1.36	0.61 – 3.01	0.45
**No (Ref)**	1		
**Use of psychoactive drugs**			
**Yes**	0.42	0.18 – 0.95	0.04*
**No (Ref)**	1		
**Number of husbands ever** **married**			
**1**	1.92	1.02 – 3.81	0.045*
**≥2 (Ref)**	1		
**Partner's age (in years)** ^+^			
**<30 years**	0.04	0.003 – 0.567	0.02*
**30 – 59 years**	0.08	0.007 – 0.776	0.03*
**≥60 years (Ref)**	1		
**Partner's Level of Education**			
**At most primary**	1.16	0.50 – 2.68	0.73
**Secondary**	1.22	0.75 – 2.01	0.43
**Tertiary (Ref)**	1		
**Partner's use of alcohol**			
**Yes**	0.41	0.25 – 0.64	<.001*
**No (Ref)**	1		
**Partner's use of psychoactive** **drugs**			
**Yes**	0.45	0.17 – 1.16	0.10
**No (Ref)**	1		
**Take decision relating to** **sex**			
**Self alone**	3.98	1.38 – 11.51	0.011*
**Husband alone**	0.31	0.19 – 0.49	<.001*
**Self and husband (Ref)**	1		
**History of sexual violence** **(before marriage)**			
**Yes**	0.08	0.45 – 0.15	<0.001*
**No (Ref)**	1		
**Respondent fight with husband**			
**Often**	0.68	0.33 – 1.39	0.29
**Sometimes**	0.80	0.46 – 1.42	0.45
**Never (Ref)**	1		

* Significant at 5%

Ref – reference category

### Factors associated with marital rape experience

The adjusted logistic model of marital rape is presented in Table 4. Women who were currently married had an increased risk of experiencing marital rape (aOR=2.68; P=0.006; CI: 1.32–5.41) compared to their divorcee counterparts.

It was observed that women who used psychoactive drugs had lower odds of experiencing marital rape (aOR-=0.42; P=0.04; CI: 0.18–0.95). Women whose husbands were less than 30 years old had a markedly lower risk of marital rape (aOR=0.04; P=0.02; CI: 0.003–0.567) com-pared with their counterparts whose husbands were aged 60 years and above. While women who took decisions on sex alone were four times more likely to suffer marital rape (aOR=3.98; P=0.01; CI: 1.38–11.51), those whose husbands took decisions on sex alone (aOR=0.31; P<0.001; CI: 0.19–0.49) were three times less likely to experience marital rape relative to their counterparts who jointly with their partners decide on sex. Women who had a previous history of sexual victimization had lower odds of marital rape experience (aOR=0.8 P=<0.001; CI: 0.045–0.15). Women's use of alcohol, number of husbands ever-married, husband's level of education and how often women fight with husbands were not observed as significant factors associated with marital rape experience (Table 4).

### Coercive strategies used for facilitating marital rape

Table 5 highlights the various forms of coercive strategies or situations that were used by the respondents' spouses to perpetrate marital rape case. Physical force (n=153, 25.9%) was the most common form of coercive strategies or situations that were used. About 84.2% (n=155) of the marital rape survivors reported the adoption of multiple coercive strategies by their spouses.

**Table 5:** Forms of coercive strategies or situations used by spouses to perpetrate marital rape experience N=184

Coercive strategies*+	Frequency (%)
**Physical force**	153 (25.9)
**Threat**	139 (23.5)
**Opportunity of ill-health**	112 (19.0)
**Verbal abuse/attack**	85 (14.4)
**Harmful substances or weapon**	67 (11.2)
**Drugs or sleep-inducing substances**	35 (5.9)

* Multiple responses were included

+ 84.2% reported the use of more than one coercive strategy.

## Discussion

The marital rape prevalence of 15.3% showed the existence and magnitude of the phenomenon in the study area. The prevalence value is not too different from what previous sentinel studies revealed. For instance, a previous study^15^ which focused on pregnant women attending antenatal clinics in two private hospitals in Lagos State revealed a prevalence of 14.2%. A prevalence of 11.6% was noted by a previous work^22^ carried out among women in two agricultural zones in Ogun State. It is to be noted that lower prevalence values have been obtained in studies which were national in outlook. Examples include a previous national survey that focused on crimes and public safety in Nigeria, which resulted in a marital rape prevalence of 2.3%^8^ and the one conducted by the NPC which revealed a prevalence of 5.5%.^23^

The marital rape cases in this study, like all cases of rape, may have been grossly under-reported. Several studies have earlier noted that the prevalence of intimate partner rape is often seriously limited by underreporting and socio-cultural factors that compel survivors to keep silent about their ordeals.^4^,^16^ Women are likely to consider cases of marital rape as normal within the socio-cultural boundary and may regard reporting such cases as drawing unnecessary attention to their private lives.^18^

The previous history of sexual victimization was an important factor of marital rape in this study. In previous studies, a history of sexual abuse in childhood or adolescence has been linked with patterns of victimization during adulthood.^1^,^24^

In this study, women who had been previously victimized sexually had a higher risk of marital rape experience. A similar trend of the result was noted in a previous study.^6^ It is possible that women with a history of childhood sexual abuse learned at a young age that forced sex is an acceptable norm to express feelings of love. Therefore, they may be more likely to tolerate such experiences within their marriages as well, excusing their husbands' behaviour as acts of love.^6^

Alcohol consumption and use of other psychoactive sub-stances by women were not observed as factors associated with marital rape in this study. This pattern of observation is contrary to what some previous studies^24^–^27^ had earlier reported about women who use alcohol and other psychoactive substances as being at a higher risk of experiencing marital rape and other violent behaviours in the hands of their husbands. A possible explanation for our own study finding could be that alcohol and psycho-active drugs used by married women may have increased their sexual capacity or made them tolerate forced sex from their husbands. Studies^28^,^29^ have noted that drugs have the potential to prevent women from acknowledging their experience as a rape victim.

The finding of our study which shows that women whose husbands indulge in the use of alcohol is contrary to what have been noted in many previous studies^26^,^27^,^29^,^30^ across different settings. Several previous studies^17^,^31^,^32^ have earlier found a relationship between a woman's risk of suffering violence and her partner's drinking habits. The finding of our study aligns with the position of some earlier researchers^33^,^34^ that alcohol use does not lead directly to marital rape, but rather other various social, cultural and personality factors may be responsible. Our finding buttresses the observation of a previous researcher that the relationship between alcohol and marital rape is complex and the data are contradictory and unclear.^13^ Also, contrary to the pattern of results in some studies^27^,^35^ our study revealed that the use of psychoactive substances by husbands was not significantly associated with marital rape. It is not impossible that some women might not have reported their husband's use of drugs because they were not aware of their husband's indulgence in such practices. Men are more likely to use psychoactive drugs such as cannabis, ecstasy, heroin, and cocaine in secluded places such as clubhouses, hideouts, and joints outside their matrimonial homes. Further evidence-based research including those focusing on men is needed to unpack the contributions of psychoactive substances to marital rape.

Our study observed that women's monthly income is not a determinant of marital rape experience. This result corroborates the finding of a previous study^16^ which noted that salary earning has no influence on the prevalence of spousal rape among women in Imo state of Nigeria. The 2013 Nigeria Demographic and Health Survey report clearly showed that there is no clear relationship between sexual violence and wealth, although women in the lowest wealth quintile are less likely to report sexual violence than women in the other quintiles.^23^ This implies that women's financial independence does not guarantee freedom from being sexually abused by husbands. While economic independence does not shield women from violence, access to economic resources can enhance women's capacity to make meaningful choices, including escaping violent situations and accessing mechanisms for protection and redress.^36^

The person who takes a decision relating to sex is an important factor in the occurrence of marital rape. Women who usually prefer to engage in sex with their husbands based on their own decision alone were at a higher risk of marital rape, while those who decide to engage in sex based on their husband's decisions alone were at a lower risk of marital rape. This is to be expected because with-out refusal there will be no need for the use of force or any other coercive strategy to indulge in forceful sexual intercourse by husbands. This finding may not be unconnected with the male dominance in sexual matters which finds its expression in most Nigerian societies^37^. Men are socialized to believe that they are superior to women and so should dominate their partners.^38^ This finding rein-forces the need to promote gender equality and sexual rights of women within Nigerian society.

This study reveals that various forms of coercive strategies were used to facilitate marital rape. These included physical force and threat. These forms of violence also characterised the context in which women in previous studies were subjected to rape by their partners.^17^,^23^ Prevention of marital rape among women should, in addition to providing general education on rape, entail equipping them with some life skills such as problem-solving, negotiation, communication skills as well as conflict-resolution skills.

## Limitation of the study

Limitations of the study include the non-inclusion of a variable like wealth index or socioeconomic status in the assessment of determinants or associated factors of marital rape; instead, only the monthly income of the respondents was assessed. Secondly, information about the number of times and the last time the respondents experienced marital rape was not requested from the participants. The information could have yielded meaningful information about point prevalence and multiple episodes of marital rape. Furthermore, the cross-sectional nature of the data limits the ability to draw causal inferences.

All assessments were based on self-reports by respondents; these are likely to be either gross underestimates or overestimates which can undermine the true prevalence of marital rape in Oyo State. Despite these limitations, data for this study comes from a large representative survey from six randomly selected LGAs in Oyo State and variables relating to personal characteristics, sexual behaviours, relationships, and partners' characteristics were tested. Also, the data provided valuable data on coercive strategies being used to facilitate marital rape.

## Conclusion

One in seven women had experienced marital rape among ever-married women in Oyo State. Psycho-social factors such as the husband's age, the person who usually takes the decisions on sex, and previous experience of sexual violence were the associated determinants of marital rape. Community-based marital rape prevention interventions such as awareness campaigns on the existence, magnitude and consequences of marital rape, advocacy for the promotion of women's sexual rights, and counselling services for survivors to enable them to overcome the psycho-social consequences that may be associated with marital rape experiences, as well as provision of rape support services, are needed to address the phenomenon.
